# From models to reality: computational estimation of acute infection prevalence from seroprevalence data—the case of *Toxoplasma gondii*

**DOI:** 10.1186/s12917-025-05098-9

**Published:** 2025-11-11

**Authors:** Elisa Fesce, Alessia Libera Gazzonis, Alessandra Barlaam, Annunziata Giangaspero, Nicola Ferrari

**Affiliations:** 1 Department of Veterinary Medicine and Animal Sciences, Università degli Studi di Milano, Lodi (LO), 26900 Italy; 2 Department of Agriculture, Food, Natural Resources and Engineering (DAFNE), University of Foggia, Foggia, 71121 Italy

**Keywords:** Incidence, Infection, Mathematical model, Modelling, Parasites, Pathogens, Prevalence, Surveillance, *Toxoplasma gondii*

## Abstract

**Background:**

The use of serological tests based on antibody detection plays a pivotal role in the definition of past exposure to pathogens and therefore monitor infection presence, guide treatment decisions, and support disease control efforts through detection and surveillance. Nevertheless, the information obtained from serological antibody tests may be incomplete, as acute infections can go unnoticed due to the delay between infection and the development of detectable antibodies. This is particularly relevant for those pathogens for which acute cases drive pathogen transmission and are associated with the onset of clinical symptoms. We therefore developed a computational framework based on mathematical models to estimate the prevalence of acute infections from serological testing to be broadly applicable to different pathogens.

**Results and conclusions:**

We showed the effectiveness of our framework and highlighted that, in addition to seroprevalence, prevalence of acute cases also depends on the recovery rate and the mean life expectancy of the population. We applied our framework to *Toxoplasma gondii*, a model pathogen for infections that are largely asymptomatic, highly prevalent in herds, and exhibit clinical signs (e.g., abortions) associated with the acute phase of a primary infection (following the acute phase, in the case of *T. gondii*). Despite the sanitary importance of diagnosing acute infections, in livestock *T. gondii* infection is usually investigated by identifying specific antibodies through serological testing, which limits our ability to predict the infection risk and the expected number of abortions. Through this worked example, we showed that our model allows for the estimation of the number of individuals in acute infection phase and prediction of the infection dynamics, providing valuable insights into disease spread and informing management strategies for the control of pathogens. Also, given the generalizability of the model proposed, it can be easily applied to different pathogens whose diagnosis relies on serological testing. Finally, to enhance accessibility, we have developed an interactive Shiny application to support the implementation and use of the framework.

**Supplementary Information:**

The online version contains supplementary material available at 10.1186/s12917-025-05098-9.

## Introduction

Infectious diseases in livestock pose substantial economic challenges due to reduced productivity, treatment costs, mortality losses and disruption of both regional and international trade in animals and animal products [1]. Additionally, they can represent a significant public health concern due to their potential to be transmitted from animals to human [2]. This underscores the need for effective surveillance, prevention, and control measures to reduce the spread of infections within animal populations [3]. Diagnostic tests are essential for monitoring the spread of infections and serological tests are among the most commonly used screening methods. Nevertheless, they are usually based on the detection of Immunoglobulin isotype G (IgG) which normally appears few weeks after infection [4], consequently indicating past exposure to the pathogens and failing to provide an accurate depiction of the current infectious state of individuals. From an epidemiological perspective, seroprevalence can be influenced not only by the number of new infections, but also by demographic and physio-pathological factors such as the natality and mortality rates or immunity loss rate. This variability can lead to misinterpretation of seroprevalence data, as similar seroprevalence values may arise from significantly different values of infection incidence.

Diagnostic tests targeting other biomarkers, such as early antibodies (IgM) and circulating DNA of the pathogen, are often suitable for detecting new cases in the acute phase of infections. However, in veterinary medicine especially, many diagnostic tests are available only as in-house protocols or are validated for use in specific host species, making large-scale application challenging [5]. Moreover, due to the brief duration of pathogen persistence in the bloodstream, testing acutely infected individuals presents significant technical challenges, and might necessitate of a large number of individuals to identify positive cases [6]. Consequently, for a considerable number of pathogens, precise estimates of the number of currently infected individuals are lacking. Yet, the timely identification of those in the acute phase of infection is crucial for implementing early and effective interventions. As acutely infected animals play a key role in transmission, accurately detecting their prevalence is essential to prevent the further spread of infection within populations. To address these issues, we propose the implementation of a computational framework to estimate the prevalence of acute infection from serological data. The tool we present is a generalised compartmental model of infectious disease transmission; it enables the explicit simulation of hidden processes, such as the occurrence of new infections, thereby allowing the inference of rates that cannot directly be estimated through epidemiological tests. Given the generalizability of the framework, it can be applied to different pathogen infections. We applied it to *Toxoplasma gondii* in herbivore intermediate hosts as a case, a pathogen whose predominantly asymptomatic infection impedes diagnosis based on symptoms alone study (box 1). *Toxoplasma gondii* is prevalent among herds and causes substantial economic losses, as infection during pregnancy induces abortions, thus underscoring the importance of accurately estimating the number of acute cases to assess the expected impact. However, its detection in herds and in the veterinary field typically relies on serological testing, making it an ideal candidate for applying our framework. Finally, to ensure the method is easily accessible, an interactive Shiny application has been developed. This application provides a user-friendly tool for computing and testing the framework. The online application allows users to easily apply and explore the method without requiring extensive technical expertise.

### Box 1: Key biological features and essential information on *Toxoplasma gondii*

**Table:** 

***Toxoplasma gondii***	
• **Life Cycle:** Involves felids as definitive hosts and homeothermic vertebrates (including humans) as intermediate hosts.	
• **Ways of infection:** horizontal transmission typically occurs through the ingestion of environmental sporulated oocysts (the infective stage), e.g. through consumption of contaminated forage by herbivore hosts. Alternatively, carnivores can become infected through the ingestion of tissue cysts. During the acute phase, the vertical transmission can occur: T. gondii can cross the placental barrier, potentially infecting the foetus or colonizing the udder and passing into the milk.	
• **Phases of infection:** the acute phase lasts about two weeks and is characterized by rapid replication, with tachyzoites invading body tissues. In the following chronic phase, the infection stages encyst in body tissues and evolve into bradyzoites, which replicate slowly and persist long-term.	
• **Symptoms in intermediate hosts:** the consequences of infection (through horizontal transmission) in immunocompetent hosts are generally mild, with most showing asymptomatic infection or mild, non-specific symptoms like fever and loss of appetite [7].	
• **Reproductive Failures:** Primary infection during pregnancy can lead to reproductive failures, including abortion in both humans and some livestock species. In sheep and goats up to 42% of aborted foetuses worldwide have been found positive for T. gondii infection [8], underscoring the importance of this pathogen as a cause of reproductive failure and its economic repercussions in the livestock industry [7].	
• **Diagnosis:** The predominantly asymptomatic nature of most T.gondii infections renders clinical diagnosis alone insufficient for detecting the pathogen in a flock, underscoring the importance of testing to establish an effective early warning system. Nevertheless, most studies rely on serological testing [9], and this approach may over-look acute infections.	

## Methods

**Fig. 1:**
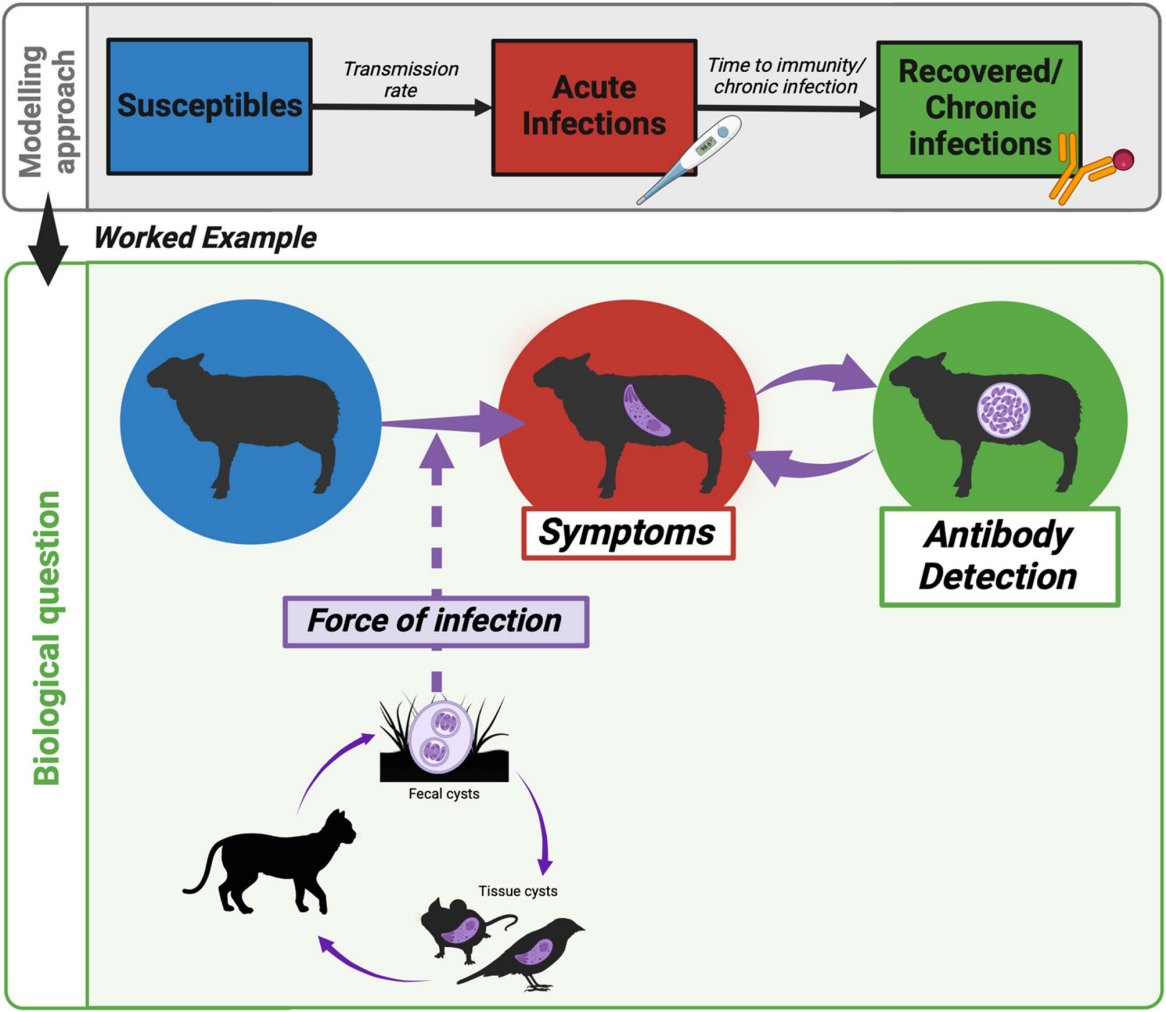
Framework scheme. Scheme of the proposed framework for estimating prevalence from seroprevalence, integrating the theoretical model with a case study. Created in https://BioRender.com

Mathematical modelling is used to estimate the prevalence of acute infections from seroprevalence data. Building on classical SIR models [10], we adapted the framework to create a Susceptible-Acute-Immune model able to simulate infection dynamics and derive the formulae to estimate prevalence. In this formulation, P_*S*_ represents the proportion of susceptible individuals; P_A_ denotes the prevalence of acute infection who are also infectious (and represents the prevalence of acute infections); and P_I_ represent individuals who have developed circulating antibodies and are therefore seropositive. The complete derivation from the original model to our framework is reported in the supplementary materials (supplementary materials text 1). From model equations, we extracted the formulae and produced the general framework to estimate the prevalence of acute infections from seroprevalence data. This framework is applicable to any pathogen that develop an acute phase followed by a clinical recovery which avoid new primary infection. After the extraction of the general formulae, we applied them to the case of *T. gondii* in a population of intermediate herbivore (Fig. 1).

### Dynamics of infection: conceptual framework to infer the incidence of new infections

#### Base model

The dynamics of infection was described by a deterministic compartmental model with continuously occurring events, according with the following system of differential equations:

1 $$\begin{aligned} &\frac{dP_S}{dt}=b-\left(\lambda\:+d\right)(1-P_A-P_I)\\&\frac{dP_A}{dt}=\lambda\left(1-P_A-P_I\right)-\left(\sigma+d+a\right)P_A\\&\frac{dP_I}{dt}=\sigma P_A-dP_I \end{aligned}$$

Where we included three main compartments representing the proportion of: susceptible individuals (P_S_), infected individuals in the acute phase of infection (P_A_), and individuals with circulating antibodies (P_I_). Individual hosts enter the system by birth as susceptible, and for the sake of simplicity, no vertical transmission is included. This modelling choice is based on the assumption that horizontal transmission represents the main route of infection maintenance in the host population. Natural mortality of hosts is included in the model (parameter *d*). For our simulations we considered a catalytic model where the force of infection $\:\lambda\:$ represents the rate at which susceptible individuals become infected, per unit of time. This choice allows the simulation of new infections independently of the contact rates between susceptible and infectious individuals when the system is at endemic equilibrium. Among possible transmission routes, it is particularly appropriate for simulating infections acquired through ingestion of environmentally persistent infectious stages, when their presence is widespread and constant, as in our worked example of *T. gondii* infection in herbivorous ruminants. This current set of equations (Eq. 1 a-c) considers environmental transmission of infection, but an extension to other routes of transmission is provided in the supplementary material (text 2) to ensure the generalisability of the framework. The passage from the acute (P_A_) to the following phase of infection (P_I_) corresponds to the recovery rate.

#### Estimate of acute infections

In a population at its endemic equilibrium, the seroprevalence of the population can be considered as a proxy of the proportion of the individuals with circulating antibodies (P_I_). Since in our set of equations, the prevalence of acute infection (P_A_) is influenced by the mean lifespan of the host individuals and the duration of the acute infection, by knowing the seroprevalence (P_I_) the death rate (d) and the recovery rate (σ), we can derive from Eq. 1c the prevalence of acute infections (P_A_) as:

 

2 $$\:{P}_{A}=\frac{d{P}_{I}}{\sigma\:}$$

A full exploration of this relationship is provided in the supplementary materials (Text 3).

Notably, in our framework, the proportion of acute infections at equilibrium depends solely on the proportion of seropositive individuals (P_I_), the death rate (d), and the recovery rate (σ), and is independent of all other parameters. It is worth highlighting that the mean death rate of the population (d) is relatively easy to estimate—especially in livestock systems, where death is often linked to decisions regarding animal productivity. Similarly, the recovery rate is typically one of the easiest parameters to estimate in non-wild settings, as it usually corresponds to the reciprocal of the mean duration of infection.

In most infections, the prevalence of acute infections (P_A_) can be directly utilised to predict the infection burden by estimating the proportion of acute cases that are likely to develop symptoms, die or require hospitalisation. In this study, we expand upon this methodology by incorporating supplementary calculations to estimate the risk of developing symptoms, with a particular focus on cases where symptomatology is conditional, such as infections that only manifest under specific circumstances (e.g., during pregnancy).

### Force of infection

Developing further Eq. 1b, we can obtain an explicit formula to estimate the force of infection ($\:\lambda\:$) as follows:

3 $$\:\lambda\:=\frac{(\sigma\:+d+\alpha\:){P}_{A}}{1-({P}_{A}+{P}_{I})}=\frac{{P}_{I}\left(\sigma\:+d+\alpha\:\right)d}{\sigma\:-{P}_{I}(d+\sigma\:)}$$

In the present study, the force of infection, where all parameters are expressed as daily rates, represents the daily rate at which susceptible individuals become infected. It follows that, at equilibrium, the individual daily probability [11] of infection $\:{r}_{A}$ is.

 

4 $$\:{r}_{A}=1-{e}^{\lambda\:}=1-{e}^{\frac{(\sigma\:+d){P}_{A}}{1-({P}_{A}+{P}_{I})}}$$

The resulting risk of infection within a given time interval (δ) is as follows:

5 $$\:{R}_{A}=1-{\left(1-{r}_{A}\right)}^{\delta\:}$$

### Work example: application to hypothetical field condition

To illustrate the practical use of the framework, we applied it to a worked example estimating the prevalence of acute *T. gondii* infection (P_A_) in a flock of intermediate herbivore hosts. Despite the complexity of *T.gondii* life cycle, which involves multiple host species, herbivore hosts are primarily infected through ingestion of environmental infective stage (oocysts) and subsequently developing chronic infection after an initial acute phase. As our worked example focuses on estimating prevalence from seroprevalence in herbivore hosts, we only considered environmental transmission via the faecal–oral route and not direct-contact infection. This assumption on environmental infection is supported by the estimates that in areas where *T. gondii* is endemically present, the burden of infective oocysts can range from 1.7 × 10⁵ to 4.7 × 10⁷ oocysts per hectare [12]. Hence, explicit modelling of infection dynamics in the definitive host was omitted as redundant for the purposes of the present work. We therefore applied the framework proposed, in which individuals in the acute phase of infection represent those undergoing tachyzoite replication, while individuals with chronic infection harbour tissue cysts composed of bradyzoites and have detectable IgG antibodies. Consequently, susceptible individuals are uninfected. In this example, the transition from the acute phase, characterised by tachyzoite replication, to the chronic phase, in which bradyzoites encyst in body tissues, corresponds to the recovery rate and development of IgG antibodies. We then simulated the dynamics of *T. gondii* in a flock of 100 individuals, and estimated (from Eq. 2) the prevalence of acute infections by assuming that the seroprevalence (P_I_) is 30%, the mean lifespan of flock individual is 5 years (d = 0.00055 day^−1^), and recovery occurs in 14 days (therefore day σ=0.071 day^−1^). Also, as *T. gondii* infections are usually asymptomatic, we assumed that there is no increase in mortality induced by the parasite (α = 0). To explore additional applications of our approach, through Eq. 5, we estimated the daily probability of becoming infected during pregnancy ${\widehat R}_A$ in our worked example, assuming that the mean duration of pregnancy in sheep is 152 days: $\widehat{R}_A=1-\left(1-r_A\right)^{152}$

From the probability of becoming infected during pregnancy ${\widehat R}_A$ , we can extrapolate the expected number of abortions ($\:\varTheta\:$) in a flock of N sheep as follows:

 

6 $$\mathrm\theta={\mathrm p}_{\mathrm s}{\mathrm R}_{\mathrm A}\mathrm N$$

Where $\:{p}_{s}$ is the proportion of acute cases that develop symptoms and N is the number of individuals in the population. In our worked example therefore, if we assume a constant probability of reproductive failure of 40% due to *T. gondii* in sheep that acquire a primary infection during pregnancy [13], and, for the sake of simplicity, that this probability remains constant. If we further assume a mean of one pregnancy per sheep per year, the expected number of abortions ($\:\varTheta\:$) in a flock of 100 sheep is:$\theta=0.4\widehat{R}_A100.$

### Shiny interface for the computational framework

To make our framework accessible, we implemented an interactive Shiny app in R, based on the formulae described above, to estimate the prevalence of acute infections from seroprevalence data. The Shiny app implements all the formulae presented in this study to estimate the prevalence of an environmentally transmitted infection in a population at equilibrium, using only serological data along with information on the average mortality rate and the duration of infection. The app is available at:

https://elisafesce.shinyapps.io/from_models_to_reality/.

This app consists of a single interactive session that allows users to calculate the prevalence of acute infections in a population. It can be applied to any infection, assuming that it is at the endemic equilibrium (i.e. the population is stable, and the infection is not of recent introduction), provided that individuals acquire lifelong immunity after recovery. The application requires only three input parameters—seroprevalence (P_I_, %), life expectancy (d, in months), and infection duration (σ, days)—and returns the expected prevalence of acute infections (P_A_).

All simulations and analyses were performed using the software R (R version 2023.09.1 + 494 (2023.09.1 + 494)), package “*deSolve*”.

## Results

**Fig. 2:**
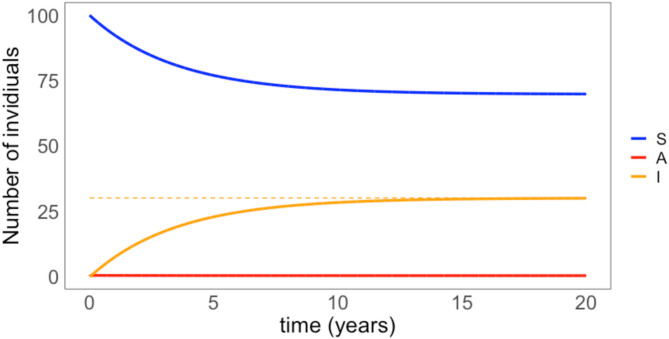
Simulated dynamics of infection. Infection dynamics of a flock of 100 individuals with a mean life expectancy of 5 years is introduced in an area infected by *T. gondii*. The blue line represents the number of susceptible individuals over time, the orange solid line represents the number of individuals with a chronic infection, and the red line represents the number of individuals with an acute infection. The orange dashed line represents the expected seroprevalence given model parameters

From Eq. 1a-c we simulated the dynamics of the *T. gondii* infection in a fully susceptible flock of 100 sheep that acquire the infection after environmental exposure (Fig. 2).

Also, if we apply Eq. 2 to an hypothetical field condition of a flock of 100 sheep, with 30% seroprevalence (*P*_*I*_), Pi=an individual mean life span of 5 years (d = 0.00055 day^−1^), assuming that the acute infection in sheep lasts for 14 days ( σ=0.071 day^−1^), we can estimate that the prevalence of *T. gondii* acute infections in the flock at the equilibrium is 0.23%. From Eq. 4 we can also derive that the force of infection ($\:\lambda\:$ )the flock undergoes to is 23.74*10^−5^ infections per day. It follows that the probability of becoming infected during pregnancy (from Eq. 5) is 3.54% and the expected number of abortions in the 100 sheep flock is 1.42 abortions/year.

## Discussion

In this work, we propose a framework to estimate the prevalence of acute infections utilising seroprevalence data as a primary source. Through rigorous mathematical modelling, we show the efficacy of this approach in accurately estimating infection prevalence. To prove our framework, we simulated a routine scenario in which pathogen presence is tested through serological methods and applied our computational approach to estimate the expected prevalence of acute infections in a flock of small ruminants exposed to environmental contamination by *T. gondii*.

Estimating the prevalence of acute infections within a population is crucial for the effective management and control of many pathogens, as it is typically the hosts that acquire a primary infection, and are in the acute phase of infection, that are at the greatest risk of developing symptoms. Despite this, several infections, in veterinary medicine in particular, often rely on serological testing as the routine method, thereby missing crucial information about the acute infection and being unable to predict its burden. Computational methods offer versatile in-silico tools that can be applied to various pathogens and epidemiological contexts in support to diagnostic testing, overcoming time and financial constraints. Furthermore, the incorporation of user-friendly interfaces, such as Shiny, serves to enhance the accessibility of these methodologies, thereby enabling their application by a wide range of users. Conversely, the development of new diagnostic tests designed to identify acute infections frequently necessitates considerable expenses and demands highly specialised technical expertise, rendering such tests impractical for routine large-scale implementation. We have therefore developed a computational approach as a viable alternative to specific diagnostic tests when the objective is to assess infection prevalence at the population level. It is important to note, however, that for the diagnosis of individual animals, the use of dedicated diagnostic tests remains the only available option. Conversely, when the focus shifts from individual diagnosis to a broader understanding of infection dynamics within a population, our framework provides a valuable and effective alternative. The findings of this study show that computational tools can be used to estimate the prevalence of acute infections from serological data, provided that the mean duration of infection and the average lifespan of individuals within the population are known. This underscores the importance of these tools in the management of infectious diseases and serves to emphasise their value as a valuable implement in such contexts. We theoretically showed that by knowing the seroprevalence of an infection indeed, we can predict the number of acute infections in a population, thereby acquiring insights into the expected number of symptomatic cases (e.g., predicting the number of abortions in the case of toxoplasmosis). This, in turn, provides valuable information on the impact of acute infections, allowing for an estimate of the associated sanitary and economic consequences of infectious diseases. Furthermore, by estimating the expected number of symptomatic cases, we can identify anomalies that signal the potential presence of other infections with similar symptoms. In addition, this approach could provide valuable insights into the efficacy of the current surveillance system, helping to assess its ability to detect and respond to emerging threats.

Toxoplasmosis serves as a prime example of this scenario, where serological methods are the prevailing standard for routine testing, yet the primary consequence of the infection—abortions—arises when the acute phase of a primary infection occurs during pregnancy. We therefore showed a practical application of our theoretical framework using *T. gondii* as a case study, where a prediction of acute infection and the number of subsequent abortions that our framework produces has practical implications for farmers, that can foresee the expected losses attributable to *T. gondii* infection. This prediction improves livestock management by providing an early warning system when the number of abortions exceeds the expected threshold. Several infectious diseases other than toxoplasmosis (e.g., chlamydiosis, brucellosis and salmonellosis) [14], can lead to abortions in livestock. Therefore, by assessing the number of abortions attributable to *T. gondii*, we can identify deviations from expected outcomes, which can signal the presence of additional infections circulating, allowing for appropriate and timely intervention.

Furthermore, from a public health perspective, although data are still limited [15, 16], there is evidence that *T. gondii* may be transmitted through unpasteurized milk, particularly caprine milk [17], implicating a possible significant role of acutely infected goats [18, 19] and underscoring the need for accurate estimation of acute infection prevalence to assess milk contamination and human transmission risk. In this context, *T. gondii* serves as an example where knowing the number of acute infections is crucial for estimating the risk of transmission and possibly preventing the spread of the disease, as precise estimates can inform interventions and risk assessments, and shows the importance of having quick and ready-to-use tools to estimate acute infections. Therefore, a framework such as the one we propose could have positive repercussions both in terms of livestock productivity and human health, since it could also be applied to human health by supporting surveillance efforts through rapid estimates of transmission risk.

Beyond the findings specific to toxoplasmosis, the proposed framework can be easily generalizable and applied to other parasitic, but also bacterial and viral, infections where estimates of acute case occurrence are scarce.

The proposed formulae rely on the standard SIR framework, which provides a simplified but broadly applicable representation of transmission dynamics, as long as the infection is characterised by distinct susceptible, infectious, and recovered phases. As such, they can be applied to a broad class of infections that are comprehensively captured by this structure—specifically, those characterised by three distinct stages: uninfected, acutely infected, and immune. Within this framework, differences between infections—such as the mode of transmission, the duration of infection, or host-specific traits—are reflected in the values of model parameters. For example, the transmission route, whether oro-fecal or respiratory, is captured by the transmission rate (β), while the average duration of infection informs the recovery rate (σ). Provided the infection follows this general SIR-like structure and the system is at equilibrium, the derived formulae apply, relying only on the death and recovery rates—parameters that are typically easier to estimate. While model extensions addressing different transmission mechanisms (environmental vs. direct transmission) are presented in the Supplementary Materials, further generalisations are also possible. For instance, the current framework assumes a homogeneous host population and does not account for the possibility that distinct groups (e.g. sex or age classes) may experience different transmission or recovery dynamics. In cases where such heterogeneities are epidemiologically relevant, model extensions involving the addition of further compartments would be necessary. Similarly, other realistic scenarios, such as infections that do not confer lifelong immunity, could be captured by incorporating a rate of immunity loss, which would return individuals to the susceptible class while depleting the immune population. However, any addition of compartments entails a reformulation of the equations used to estimate prevalence. This process may potentially preclude an analytical solution for the equilibria. This underscores a key feature of mathematical models: they are necessarily simplified representations of reality capturing essential features of the system.

In this framework, recovered individuals gain immunity, do not undergo recrudescence, and cannot be reinfected. For instance, it could provide useful insights for infections such as *Besnoitia besnoiti*, a Toxoplasmatinae parasite infecting cattle, where detecting acute cases of infection in addition to those in the chronic phase, facilitates the development of targeted control strategies [20]. During the acute phase, infected animals may exhibit non-specific signs (fever, ocular and nasal discharge, lymphadenitis, edema, and orchitis) and experience a decline in both reproductive and productive performances. Specific serological tests, such as in-house Avidity ELISA and IgM targeting tests, have been proposed for detecting acute infections and identifying animals that may test negative on standard IgG tests [21], but they cannot be routinely employed in diagnostics.

Stepping beyond the field of parasitology and Toxoplasmatinae parasites, hepatitis E virus (HEV) arises as another noteworthy infection where the application of a computational framework to estimate the prevalence of acute infections could be beneficial. Anti-HEV antibodies are indeed frequently detected on wild boar hunted for human consumption; however, it is the handling or consumption of viremic individuals that poses a significant threat for human health. Furthermore, field investigations have highlighted substantial discrepancies between seroprevalence and RNA-based prevalence estimates (e.g [22]. and others), likely reflecting differences in diagnostic protocols. This situation exemplifies how a theoretical framework can serve as an auxiliary baseline for interpreting and comparing empirical data, providing an estimate of the expected acute infection prevalence in wild boars can improve our understanding of the real risk of infection for humans, can aid in planning further studies, as well as improving sampling design, diagnostic testing and disease risk assessment.

Overall, the essential mathematical structure of our model is a key strength of our framework, making it both easy to apply and extend to different situations. More complex and realistic models can be built to investigate infection mechanisms, but they require a substantial amount of high-quality data, which can be difficult and costly to collect, if not impossible. Furthermore, increased complexity in model formulation often corresponds to greater difficulty in interpreting the results. In contrast, simpler models can be valuable for disentangling unclear processes or analyzing specific aspects of infection by leveraging the data we currently have or that already exist. In our work, the simple structure requires limited and easily accessible data. Given that the model proposed is based on an estimate of the force of infection, it does not strictly depend on a single specific mechanism – like could be the encounter rates with infectious stages (bradyzoite vs. tachyzoite) or the consumption of a single food source (vegetable vs. meat) – making it easy to adapt to various specific context and infections. On the one hand, the straightforward and basic structure of the model we propose comes at the expense of the incorporation of processes such as immune-loss or age class differences, which may result in an oversimplification of infectious processes. On the other hand, this foundational mathematical framework represents its key advantage, allowing for easy application and adaptation across various contexts and infections.

## Conclusions

In conclusion, we propose a computational framework designed to estimate prevalence of acute infections from seroprevalence estimates, demonstrating its application through a case study of *T. gondii* infection in a small ruminant flock. Through our worked example we highlighted the effectiveness of the proposed framework in generating realistic estimates for the number of acute infections, as well as in forecasting the expected yearly number of abortions. We therefore showed that this framework can serve as a valuable complement to conventional diagnostic tests by providing essential information on the number of acute infections – information that is epidemiologically crucial for assessing transmission risk and for quantifying the expected burden of disease, particularly in infections where clinical manifestations occur primarily during the acute phase. Therefore, our approach offers a practical and scalable tool for improving disease surveillance and informing targeted intervention strategies. Also, the structure of our model allows for the application of the proposed framework to various other parasitic and infectious diseases.

## Supplementary Information

Supplementary Material 1

## Data Availability

Data sharing is not applicable to this article as no datasets were generated or analysed during the current study, the modelling framework is fully described in the methods for the reproducibility of the results.
