# Supplementary material for: From models to reality: computational estimation of acute infection prevalence from seroprevalence data—the case of Toxoplasma gondii
Source: BMC Vet Res. 2025 Nov 11;21:657. doi: 10.1186/s12917-025-05098-9 (PMC12607226; doi:10.1186/s12917-025-05098-9)
Supplement: Supplementary file 1 — Supplementary Material 1 [file 12917_2025_5098_MOESM1_ESM.pdf]

From models to reality: computational estimation of acute infection prevalence from seroprevalence data—the case of toxoplasma gondii

Elisa Fesce[1], Alessia Libera Gazzonis[1], Alessandra Barlaam[2], Annunziata Giangaspero[2], Nicola Ferrari[1]

1. Department of Veterinary Medicine and Animal Sciences, Università degli Studi di Milano, 26900 Lodi (LO), Italy
2. Department of Science of Agriculture, Food and Environment (DAFNE), University of Foggia, 71121 Foggia, Italy

***Corresponding Author.** elisa.fesce@unimi.it

Supplementary information:

Text 1. Original Equations and Model Construction

System 1: base model - Environmental Route of Transmission

The base model was described by the following system of differential equations.

$$\frac{dS}{dt} = b(S + A + I) - (\lambda + d)S \qquad \textit{Eq.1a.S1}$$

$$\frac{dA}{dt} = \lambda S - (\sigma + d + \alpha)A \qquad \textit{Eq.1b.S1}$$

$$\frac{dI}{dt} = \sigma A - dI \qquad \textit{Eq.1c.S1}$$

Where we included three main compartments: susceptible individuals (S), infected individuals in the acute phase of infection (A), (in the worked example those individuals with tachyzoites replication), and individuals with circulating antibodies (I), (in the worked example those individuals with tissue cysts composed by bradyzoites). Individual hosts enter the system by birth as susceptible, and no vertical transmission is included. Natural mortality of hosts is included in the model (parameter *d*). For our simulations we considered a catalytic model where the force of infection λ represents the rate at which susceptible individuals become infected, per unit of time. Notably, in this scenario transmission occurs due to environmental contamination by free living

stages of the parasite (or general pathogen environmental contamination), and therefore does not require infectious individuals to sustain the infection. This current set of equations (Equations 1 a.S1, b.S1 and c.S1) considers environmental transmission of infection, but an extension to other routes of transmission is provided in the supplementary material to ensure the generalisability of the framework. The passage from the acute (A) to the following phase of infection (I) corresponds to the recovery rate.

To predict the prevalence of acute infections from seroprevalence data in a population at its endemic equilibrium, we adapted our base SAI model as follows: at first, we expressed the whole number of hosts (N) as the sum of the number of individuals in the three compartments as follows:

$$N = S + A + I$$

Notably N is constant, given the assumption to be at equilibrium.

Although the number of acute infections (A, equation 1b.S1) depends on time, as we assumed the system to be at equilibrium, equations 1a.S1–1c.S1 yield the expression $A = \frac{N\alpha}{(b-d)}$. This highlights a relationship between the number of acute infections (A), the population size (N), and the model parameters (parasite-induced mortality, α, natural death rate, d, and birth rate, b). According with [1], we then expressed the prevalence of individuals in the acute phase (P_A) as:

$$P_A = \frac{A}{N}$$

And the prevalence of individuals with circulating antibodies (P_I) as

$$P_I = \frac{I}{N}$$

It follows that the proportion of susceptible individuals may be expressed as

$$P_S = 1 - (P_A + P_I)$$

The modification of the model gives rise to the system of differential equations presented in the main text. The specific equations are as follows:

$$\frac{dP_S}{dt} = b - (\lambda + d)(1 - P_A - P_I) \qquad \textit{Eq.2a.S1}$$

$$\frac{dP_A}{dt} = \lambda(1 - P_A - P_I) - (\sigma + d + \alpha)P_A \qquad \textit{Eq.2b.S1}$$

$$\frac{dP_I}{dt} = \sigma P_A - dP_I \qquad \textit{Eq.2c.S1}$$

Equilibria: base model equilibria

We calculated the system's equilibrium by setting all derivatives to zero, resulting in the following equations:

$$P_A = \frac{d}{\sigma} P_I$$

$$P_I = \frac{\lambda\sigma}{\lambda(d+\sigma)+(\sigma+d+\alpha)d}$$

Notably, the solutions presented here can be used to directly estimate infection prevalence from known parameters. However, the force of infection remains a hidden process, for which no direct field measurement is typically available.

Text 2. Model Extension - Direct Contact Transmission

To broaden the applicability of our framework, we generalised the original formulation—initially developed for environmentally transmitted infections—by incorporating a direct transmission component. Specifically, we introduced a transmission term that represents the rate of new infections arising from contacts between susceptible and acutely infected individuals ($\beta \frac{SA}{N}$). This contrasts with the environmental transmission model, in which the transmission term depends solely on environmental contamination and the number of susceptible hosts (λS).

System 2: transmission frequency dependent

$$\frac{dS}{dt} = b(S + A + I) - \beta \frac{SA}{N} - dS \qquad \textit{Eq. 1a.S2}$$

$$\frac{dA}{dt} = \beta \frac{SA}{N} - (\sigma + d + \alpha)A \qquad \textit{Eq. 1b.S2}$$

$$\frac{dI}{dt} = \sigma A - dI \qquad \textit{Eq. 1c.S2}$$

Where N is the total number of individuals in the population, S are the susceptible individuals, A the infected individuals in the acute phase of infection and I are the individuals who have developed circulating antibodies (I). β here represent the transmission rate of the infection and depends on the transmissibility of infection chosen, b and d are the birth and death rate of the

population respectively; σ is the recovery rate (i.e. $\sigma = \frac{1}{duration\ of\ infection}$) and α is the mortality rate induced by the pathogen (0 if the pathogen do not induce any mortality).

From Equations 1a.S2, 1a.S2 and 3a.S2 we can derive:

$$\frac{dP_S}{dt} = b - \beta P_A P_S - \mathrm{d}(1 - P_A - P_I) \quad \textit{Eq. 2a.S2}$$

$$\frac{dP_A}{dt} = \beta P_A P_S - (\sigma + d + \alpha)P_A \quad \textit{Eq. 2b.S2}$$

$$\frac{dP_I}{dt} = \sigma P_A - dP_I \quad \textit{Eq. 2c.S2}$$

And consequently:

$$P_A = \frac{dP_I}{\sigma} \quad \textit{Eq. 3.S2}$$

And

$$\beta = \frac{(\sigma+d+\alpha)}{(1-P_A-P_I)} = \frac{\sigma(\sigma+d+\alpha)}{(\sigma-dP_I-P_I)} \quad \textit{Eq. 4.S2}$$

It follows that the, at equilibrium, the daily probability of infection r_A given a specific transmission rate β is:

$$r_A = 1 - e^{\beta} = 1 - \mathrm{e}^{\frac{\sigma(\sigma+d+\alpha)}{(\sigma-dP_I-P_I)}} \quad \textit{Eq. 5.S2}$$

System 3: transmission density dependent

Analogously, the formulae can be extended to infections with density-dependent direct transmission.

$$\frac{dS}{dt} = b(S + A + I) - \beta SA - dS \quad \textit{Eq. 1a.S3}$$

$$\frac{dA}{dt} = \beta SA - (\sigma + d + \alpha)A \quad \textit{Eq. 1b.S3}$$

$$\frac{dI}{dt} = \sigma A - dI \quad \textit{Eq. 1c.S3}$$

Where N is the total number of individuals in the population, S are the susceptible individuals, A the infected individuals in the acute phase of infection and I are the individuals who

have developed circulating antibodies (I). β here represent the transmission rate of the infection and depends on the transmissibility of infection chosen, b and d are the birth and death rate of the population respectively; σ is the recovery rate (i.e. $\sigma = \frac{1}{duration\ of\ infection}$) and α is the mortality rate induced by the pathogen (0 if the pathogen do not induce any mortality).

From Equations 1a.S3, 1b.S3 and 3c.S3 we can derive:

$$\frac{dP_S}{dt} = b - \beta P_A(1 - P_A - P_I) - \mathrm{d}(1 - P_A - P_I) \qquad \textit{Eq. 2a.S3}$$

$$\frac{dP_A}{dt} = \beta P_A(1 - P_A - P_I)N - (\sigma + d + \alpha)P_A \qquad \textit{Eq. 2b.S3}$$

$$\frac{dP_I}{dt} = \sigma P_A - dP_I \qquad \textit{Eq. 2c.S3}$$

And consequently:

$$P_A = \frac{dP_I}{\sigma} \qquad \textit{Eq. 3.S3}$$

And

$$\beta = \frac{(\sigma+d+\alpha)}{(1-P_A-P_I)N} = \frac{\sigma(\sigma+d+\alpha)}{(\sigma-dP_I-P_I)N} \qquad \textit{Eq. 4.S3}$$

It follows that the, at equilibrium, the daily probability of infection r_A given a specific transmission rate β is:

$$r_A = 1 - e^{\beta} = 1 - \mathrm{e}^{\frac{\sigma(\sigma+d+\alpha)}{(\sigma-dP_I-P_I)N}} \qquad \textit{Eq. 5.S3}$$

Note that equation 2c, from which we will derive the prevalence given the seroprevalence, and equation 3 do not change in any of the scenario proposed (i.e. Eq. 2c.S1 is equal to Eq. 2c.S2 and Eq. 2c.S3; and Eq. 3.S1 is equal to Eq. 3.S2 and Eq. 3.S3.).

Text 3. Supplementary results: Impact of host lifespan and infection duration on acute infection prevalence

Given the dependence of the expected prevalence of acute infections (P_A) on the mean lifespan of the host individuals and the duration of acute infection (from equation 3a in the main text), we explored expected number of acute infections as a function of seroprevalence for different life expectancies and duration of infection.

The investigation into the impact of varying parameters on the relationship between prevalence of acute cases and seroprevalence (equation 3a in the main text) revealed a linear relationship between the expected prevalence of acute infections (P_A) and seroprevalence. Simulations with diverse values of duration of infection and host life spans shows that the rate of increase is positively influenced by longer host lifespan and longer duration of acute infection (Figure 1). The expected prevalence of acute infections (P_A) tends to remain low (never exceeding 9.37% in all simulations performed). Especially in cases of short-term infections (i.e. 5 and 10 days), the prevalence P_A turns out to be 0.26% and 0.52% respectively (Figure 1A). For long life expectancy of the flock as a mean life expectancy of 9 and 11 years, the P_A is 0.4% and 0.33% respectively (Figure 1B). Furthermore, an increase in the duration of infection affects more the prevalence of acute infections than a decrease in life expectancy.

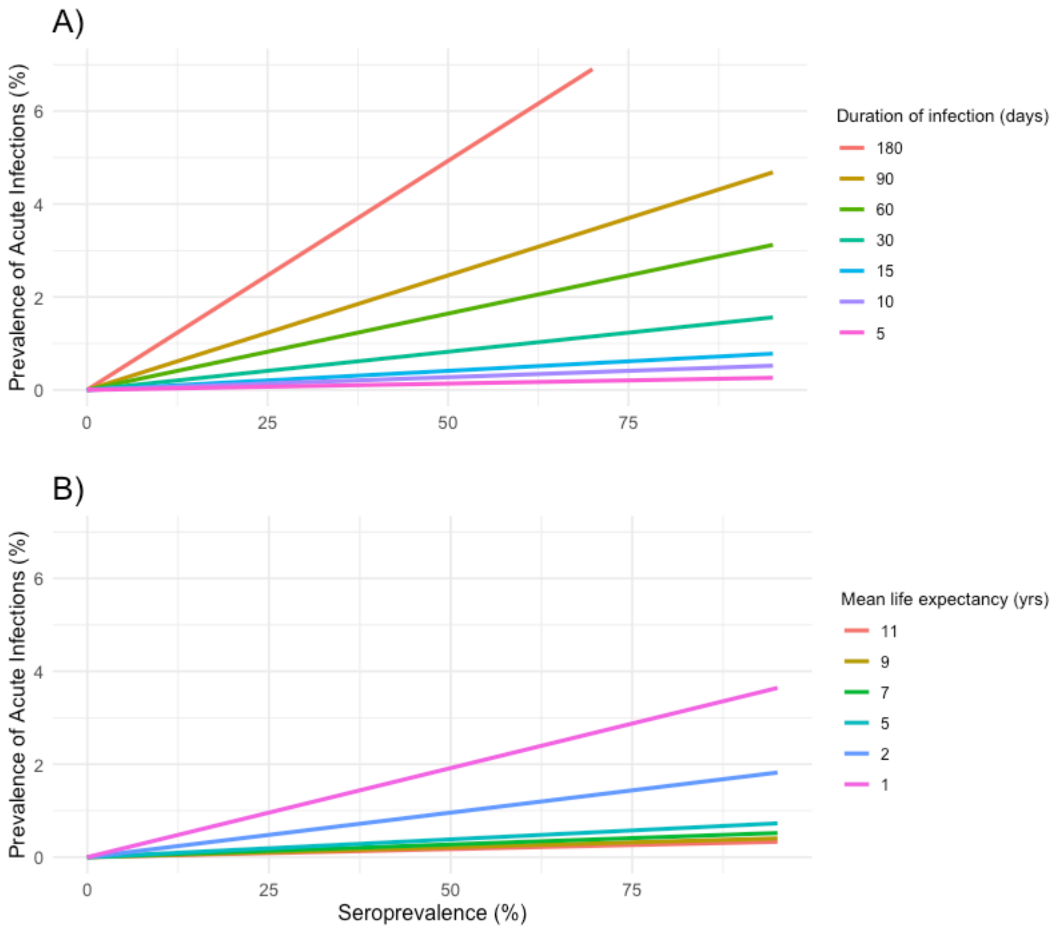

Figure 1: Expected prevalence. Expected prevalence of acute infections in relation to the seroprevalence at equilibrium. In panel A the expected prevalence of acute infections is explored for different hypothetical durations of the infection (in this set of simulations the mean life span was kept constant to 5 years). In panel B, the expected prevalence of acute infections is explored

for different hypothetical life span flock individuals (duration of infection was kept constant to 14 days).

Supplementary references:

1. Ferrari, N., Rosà, R., Lanfranchi, P. & Ruckstuhl, K. E. Effect of sexual segregation on host–parasite interaction: Model simulation for abomasal parasite dynamics in alpine ibex (Capra ibex). *Int J Parasitol* **40**, 1285–1293 (2010).
